# Intraoperative Foot Positioning During Percutaneous Calcaneal Fixation: A Technical Note

**DOI:** 10.7759/cureus.43147

**Published:** 2023-08-08

**Authors:** Shounak Taywade, Aditya L Kekatpure, Abhiram Awasthi, Ankur Salwan, Gajanan Pisulkar

**Affiliations:** 1 Department of Orthopaedic Surgery, Jawaharlal Nehru Medical College, Datta Meghe Institute of Higher Education and Research, Wardha, IND; 2 Department of Orthopedic Surgery, Jawaharlal Nehru Medical College, Datta Meghe Institute of Higher Education and Research, Wardha, IND

**Keywords:** calcaneal fracture, fluoroscopy, lithotomy stirrup, percutaneous fixation, patient positioning

## Abstract

Percutaneous screw fixation is a good modality for operative management of extra-articular and some intra-articular fractures of the calcaneum amenable to closed reduction. Tongue-type calcaneal fractures with a dislocated posterior facet are usually treated with percutaneous fixation. When treating calcaneal fractures with substantial soft tissue compromise, particularly open fractures, percutaneous reduction techniques are crucial. They also provide patients with local or systemic contraindications to open reduction with a therapeutic option. We describe the intraoperative positioning of the foot using a lithotomy stirrup during percutaneous fixation of the calcaneal fractures with minimum manipulation of the foot and C-arm and consistent imaging.

## Introduction

The calcaneum is the most commonly injured tarsal bone [1]. Displaced calcaneal fractures require surgical intervention. It can be done open or closed. Even though open reduction is the gold standard, there is an increased risk of postoperative wound dehiscence due to precarious blood supply [2]. In certain varieties of calcaneal fractures, close reduction and percutaneous fixation are viable alternatives with the added advantage of less soft tissue injury. For intraoperative assessment of reduction and articular congruity, lateral and axial views are required. With the patient in a lateral position, obtaining intraoperative fluoroscopic axial views is cumbersome [2].

It requires manipulation of the foot by the surgeon, and even after that, an accurate axial view is not always possible. Even while doing percutaneous fixation with CC screws, it becomes difficult to do surgery and take intraoperative views simultaneously [3]. With this technical note, we offer an alternative patient positioning using a lithotomy stirrup at the operation table end. In our series, we found this technique to be helpful and less cumbersome.

## Technical report

Patient positioning

**Figure 1:**
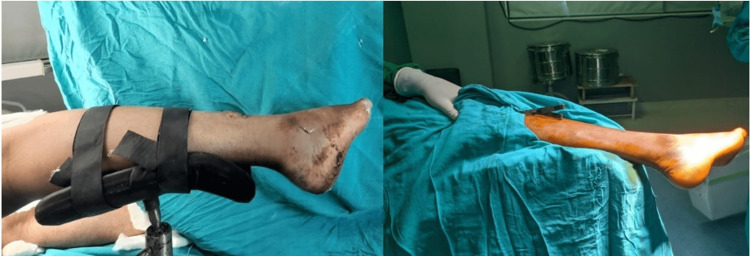
Intraoperative positioning of the patient with a lithotomy stirrup attached to the OT table with the foot hanging off the stirrup

The patient is taken in a lateral position after the application of a tourniquet. The affected leg is held at the end of the operative table over a lithotomy stirrup fixed at the end of the table. The leg is secured over the stirrup with the help of Velcro straps, and the foot hangs over the lithotomy base plate. The ipsilateral knee is supported over a pillow. The bony prominences of the contralateral limb are well-padded and protected, and the limb kept hanging off the stirrup, as shown in Figure 1.

C-arm positioning

**Figure 2:**
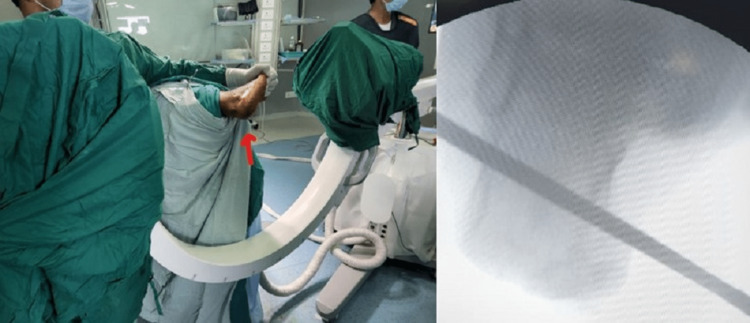
Intraoperative positioning of the C-arm for an axial view of the calcaneum

**Figure 3:**
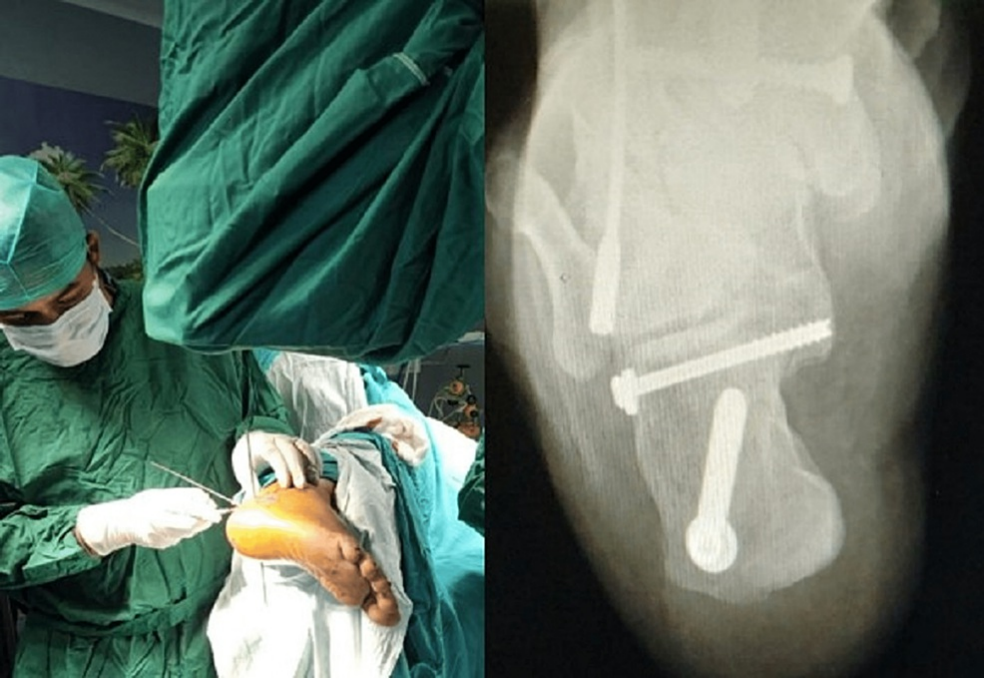
Intraoperative positioning of the patient with guidewires for screws in-situ and intraoperative post-fixation axial view of the calcaneum in the proposed positioning of the patient

The C-arm is positioned at the bed end and brought in from the contralateral side, and intraoperative fluoroscopy views are obtained for the fixation. Figure 2 illustrates the ease of obtaining the required fluoroscopic views (lateral and axial) throughout the surgery. This technique makes the C-arm positioning with screw positioning and the final axial shots easy to assess (Figure 3).

An intraoperative lateral view of the calcaneum can be easily achieved without much manipulation of the foot. There is no need to even move the C-arm after fixing it in position, and a true lateral view of the foot can be achieved without any effort. While operating in the technique advised by us, intraoperative percutaneous screw fixation can be done without any difficulty.

Advantages

For easy manipulation of the foot for intraoperative imaging under the C-arm, less intraoperative exposure to radiation, and a perfect axial view of the foot during surgery without any manipulation, we have tabulated the benefits of the proposed position in Table 1.

**Table 1:** Advantages of the proposed position

Intraoperative parameters	Traditional positioning	Proposed positioning advantage
Patient position	Lateral on a normal operation table with the foot hanging outside the table	Lateral on lithotomy support gives ease of positioning and intraoperative fluoroscopy
Intraoperative fluoroscopic axial and lateral views	Difficult to obtain and cumbersome with the repeated movement of the C-arm	Easier to obtain with less movement of the C-arm
Fluoroscopy exposure	More	Less
Fracture manipulation and temporary stabilization	Difficult to simultaneously manipulate and visualize under the C-arm	These difficulties can be overcome with this approach
Application of bilateral uniplanar distractor	Difficult	Easier and time-saving

## Discussion

When fixing fractures of the calcaneus, especially intra-articular fractures, high-quality intraoperative C-arm images are very crucial. Lateral and axial views of the calcaneum and the so-called anteromedial “constant” fragment can be visualized fluoroscopically with the Harris view, allowing for precise screw placement and preventing the penetration of screws into the medial neurovascular structures. A good axial view of the calcaneus also ensures that the heel is aligned correctly. The Harris view can be used to evaluate subtalar joint displacement, angulation of the tuberosity fragment, increase in heel width, and residual calcaneal varus after reduction in calcaneal fractures. This view is obtained by placing the X-ray source posterior to the heel and angling it caudally by about 45° about the long axis of the foot. Different surgeons have different methods of obtaining axial views of the calcaneum in the operating room.

According to Marsh et al. [3], the C-arm base should be positioned at the foot end of the bed, in front of the surgeon, and at a 45° angle to the patient’s axis. The X-ray source is arched beneath the table until the beam is parallel to the ground to produce a Harris view for percutaneous screw fixations. A two-C-arm approach for calcaneal fixation was published by Abousayed et al. [4]. The technique involves placing the patient in a lateral decubitus posture, using a conventional C-arm for lateral views and a mini C-arm with the X-ray source slightly posterior to the calf for variable-angle Harris views. According to Geerling et al. [5], after initial reduction and fixation with the use of ordinary fluoroscopy, the use of a C-arm-based three-dimensional imaging system led to the adjustment of screw placement in 41% of instances, although at a price of somewhat longer operating periods.

## Conclusions

In our experience, we have found that the proposed foot positioning technique helps ease obtaining the required intraoperative fluoroscopic views. This technique is reproducible and less time-consuming. The number of intraoperative fluoroscopic views and exposure to surgeons is also reduced if the technique is used. The number of assistants required for surgery is also less as compared to traditional foot positioning techniques. In this study, there are a couple of limitations in terms of being a single-surgeon, single-center study. Also, we have not compared the proposed positioning with the traditional approach in terms of operative time and fluoroscopy exposure. We plan to study these parameters in our future study. This technique can be a useful alternative for the percutaneous fixation of calcaneal fractures.
